# Context Dependent Role of Type 2 Innate Lymphoid Cells in Allergic Skin Inflammation

**DOI:** 10.3389/fimmu.2019.02591

**Published:** 2019-11-06

**Authors:** David A. Rafei-Shamsabadi, Christoph S. N. Klose, Timotheus Y. F. Halim, Yakup Tanriver, Thilo Jakob

**Affiliations:** ^1^Allergy Research Group, Department of Dermatology, Medical Center-University of Freiburg, Faculty of Medicine, University of Freiburg, Freiburg, Germany; ^2^Laboratory of Innate Immunity, Department of Microbiology, Infectious Diseases and Immunology, Charité-Universitätsmedizin Berlin, Berlin, Germany; ^3^CRUK Cambridge Institute, University of Cambridge, Cambridge, United Kingdom; ^4^Institute of Medical Microbiology and Hygiene, University Medical Center Freiburg, Freiburg, Germany; ^5^Department of Internal Medicine IV, University Medical Center Freiburg, Freiburg, Germany; ^6^Experimental Dermatology and Allergy Research Group, Department of Dermatology and Allergology, University Medical Center Giessen, Justus Liebig University Giessen, Giessen, Germany

**Keywords:** innate lymphoid cells, allergic contact dermatitis, atopic dermatitis, counter regulation, immune crosstalk

## Abstract

The discovery of innate lymphoid cells (ILC) has profoundly influenced the understanding of innate and adaptive immune crosstalk in health and disease. ILC and T cells share developmental and functional characteristics such as the lineage-specifying transcription factors and effector cytokines, but importantly ILC do not display rearranged antigen-specific receptors. Similar to T cells ILC are subdivided into 3 different helper-like subtypes, namely ILC1-3, and a killer-like subtype comprising natural killer (NK) cells. Increasing evidence supports the physiological relevance of ILC, e.g., in wound healing and defense against parasites, as well as their pathogenic role in allergy, inflammatory bowel diseases or psoriasis. Group 2 ILC have been attributed to the pathogenesis of allergic diseases like asthma and atopic dermatitis. Other inflammatory skin diseases such as allergic contact dermatitis are profoundly shaped by inflammatory NK cells. This article reviews the role of ILC in allergic skin diseases with a major focus on ILC2. While group 2 ILC are suggested to contribute to the pathogenesis of type 2 dominated inflammation as seen in atopic dermatitis, we have shown that lack of ILC2 in type 1 dominated contact hypersensitivity results in enhanced inflammation, suggesting a regulatory role of ILC2 in this context. We provide a concept of how ILC2 may influence context dependent the mutual counterbalance between type I and type II immune responses in allergic skin diseases.

## Introduction

**Figure 1:**
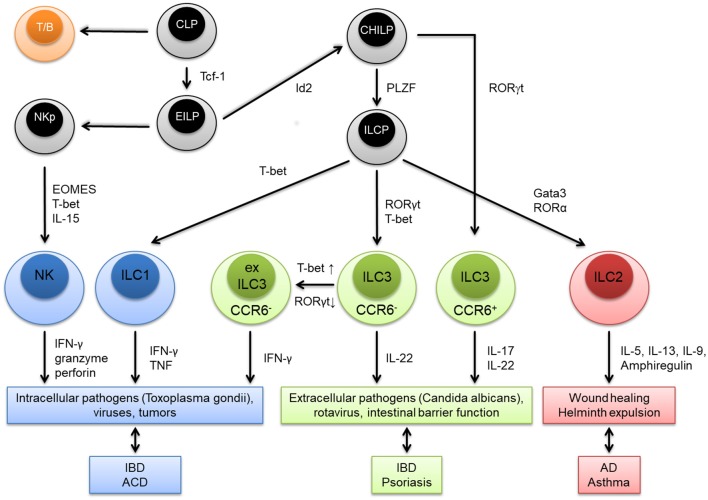
Graphical summary of innate lymphoid cell (ILC) subtypes. ILC as well as T and B lymphocytes (T/B) derive from a common lymphoid precursor (CLP). The CLP gives rise to an early innate lymphoid precursor (EILP) that expresses the transcription factor (TF) T-cell factor 1 (Tcf-1). From this point natural killer (NK) cells develop via a NK precursor (NKp) and upregulate the TFs eomesodermin (EOMES) and T-box transcription factor TBX21 (T-bet). The helper-like ILC lineage derives from an Id2 expressing common helper-like ILC progenitor (CHILP) from which C-C chemokine receptor type 6 positive (CCR6^+^) ILC3 can directly evolve depending on the expression of RAR-related orphan receptor (ROR)γt. All the remaining helper-like ILC subtypes, namely ILC1, ILC2, and ILC3, evolve from an innate lymphoid cell precursor (ILCP) which expresses the TF promyelocytic leukemia zinc finger (PLZF). CCR6^−^ ILC3 can adapt a more ILC1-like phenotype by downregulating RORγt and upregulating T-bet. These cells are called ex ILC3. Production of their marker cytokines attributes certain physiological and pathological roles to the particular ILC subtype. IBD, inflammatory bowel disease; ACD, allergic contact dermatitis; AD, atopic dermatitis.

Innate lymphoid cells (ILC) are innate immune cells of the lymphoid lineage, which have a similar functional diversity as T cell subsets based on the developmental dependency on lineage-specifying transcription factors and effector functions. Like T and B lymphocytes, all ILC derive from a hematopoietic stem cell-derived common lymphoid precursor (CLP) cell in the bone marrow (Figure 1). The CLP gives rise to an early innate lymphoid precursor (EILP) that expresses the transcription factor (TF) T-cell factor 1 (Tcf-1). From this branching point natural killer (NK) cells develop via a NK precursor (NKp) and by upregulating the TFs eomesodermin (EOMES) and T-box transcription factor TBX21 (T-bet). The other branch develops into an Id2 expressing common helper-like ILC progenitor (CHILP). C-C chemokine receptor type 6 positive (CCR6^+^) ILC3 can directly evolve form the CHILP depending on the expression of RAR-related orphan receptor (ROR)γt. All the remaining helper-like ILC subtypes, namely ILC1, ILC2, and ILC3, evolve from an innate lymphoid cell precursor (ILCP) which expresses the TF promyelocytic leukemia zinc finger (PLZF). CCR6^−^ ILC3 can adapt a more ILC1-like phenotype by downregulating RORγt and upregulating T-bet. These cells are called ex ILC3. Production of their marker cytokines attributes certain physiological and pathological roles to the particular ILC subtype (Figure 1). Effector ILC can be classified into three interleukin-7 receptor positive (IL-7R^+^) helper-like ILC groups (ILC1-3) and one IL-7R^−^ cytotoxic ILC group (NK cells) (1–3). More recently, several groups have also identified IL-10 secreting ILC with proposed regulatory functions (4–6). Helper-like ILC and NK cells are mainly populated at barrier surfaces like the skin, gut, and the respiratory tract, although significant numbers can be detected in secondary and tertiary lymphoid organs in homeostasis and disease (7). Besides the bone-marrow, alternative sites of development exist, such as secondary lymphoid organs or even non-hematopoietic organs such as the gut (8–10). While ILC development continues throughout life, it is known that some ILC lineages are long-lived, and seed their designated tissues early in embryogenesis as demonstrated by parabiosis experiments in mice that show only little replenishment of helper-like ILC from the bone marrow in later life (11–13). Although some helper-like ILC express homing receptors for certain tissues these cells are mainly thought to proliferate on site under proinflammatory conditions (7, 14). Given their localization at barrier surfaces ILC perfectly serve as sensors for danger signals but also allergens and subsequently mount early immune responses by rapid cytokine production. They can act as initiators of the adaptive immune response by crosstalk with dendritic cells and T cells finally shaping full blown type 1, 2, or 3 immune responses [reviewed in (15)]. This review highlights the pathogenic role of ILC in the allergic skin diseases with a main focus on ILC2.

## ILC Classification and Plasticity

### NK Cells and ILC1

NK cells are considered the innate counterpart of memory CD8^+^ T cells. They share similar functions such as cytotoxicity and interferon-γ (IFN-γ) production and both express the transcription factors Eomes and T-bet. ILC1 on the other hand closely resemble T_H_1 cells. Both express and depend on T-bet but lack EOMES and produce IFN-γ (16–19). NK cells and ILC1 are involved in protecting the organism against pathogens, viruses and tumors (16, 20, 21). Intraepithelial ILC1 can be found in Crohn's disease patients and contribute as a proinflammatory IFN-γ-producing population in an anti-CD40-induced colitis model in mice (22). NK cells are suggested to be important in enhancing inflammatory responses in a hapten based contact hypersensitivity mouse model and human allergic contact dermatitis (23, 24). Taken together these cell types are mainly involved in mounting a type 1 immune response.

### ILC2

ILC2, like T_H_2 cells, highly express the transcription factor GATA3 and produce type 2 cytokines including interleukin-5 (IL-5), IL-13 and the epidermal-growth-factor-like molecule amphiregulin (7). ILC2 mediate pathology in a mouse model of atopic dermatitis and promote wound healing in an IL-33-dependent manner (25, 26). ILC2 promote type 2 driven immune responses by promoting T_H_2 differentiation of naïve CD4^+^ T cells through production of IL-13, and by expression of MHC class II on their cell surface induce T cell priming (27–29). In addition, the inducible T-cell costimulatory (ICOS) molecule is highly expressed on ILC2 regulating their activation status and proliferation (30, 31). Moreover, activated ILC2 can express the TNF receptor superfamily ligand OX40L, which promotes local T_H_2 cell proliferation and adaptive type 2 inflammation (32). Increased ILC2 numbers are linked to human allergic airway and skin diseases like allergic asthma atopic dermatitis (25, 33–36). Thus, type 2 immune responses are profoundly shaped by ILC2.

### ILC3

ILC3 share RORγt expression with T_H_17 cells and can produce IL-17 and IL-22 thereby helping the organism to fight against bacteria and fungi and viruses, such as *Citrobacter rodentium, Salmonella enterica, Candida albicans*, and rotavirus (2, 7, 37–41). There are ILC3 expressing the chemokine receptor CCR6 which comprise lymphoid-tissue-inducer (LTi) cells and can be CD4^+^ or CD4^−^. These cells are crucially important in the embryonic development of many lymphoid organs, whereas in adult mice they reside mainly in cryptopatches of the intestine with low proliferation (42–45). In mice, CCR6^−^ ILC3 can express natural killer cell receptor such as NKp46 (NCR^+^ ILC3), loose RORγt expression and upregulate T-bet, finally leading to IFN-γ production (46–50). These “ex-RORγt^+^ ILC3” closely resemble ILC1. A large population of ILC3 can be found in the intestine where they are essential for maintaining barrier integrity and immunologic tolerance to commensal bacteria of the gut (51–53). IL-17 producing ILC3 are proposed to be involved in plaque formation in a psoriasis mouse model based on the topical application of the Toll-like receptor 7 (TLR7) agonist imiquimod (54). Finally, elevated numbers of ILC3 are found in blood and affected skin of psoriasis patients (55–57). Given this data ILC3 are part of type 3 immune responses and intestinal immunopathology.

## Role of ILC in Atopic Dermatitis

**Figure 2:**
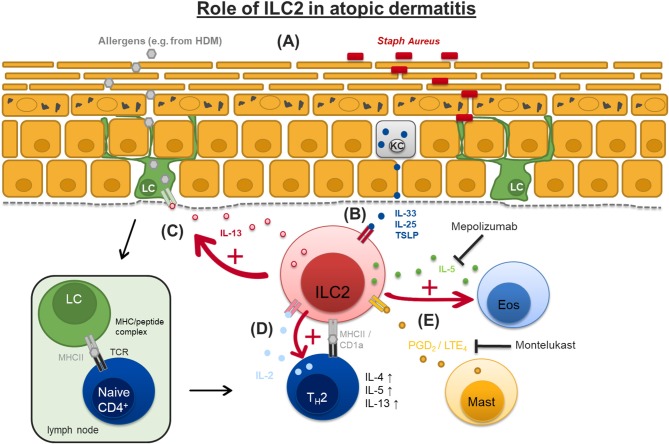
Suggested pathogenic role of ILC2 in atopic dermatitis. **(A)** Loss-of-function-mutations in the gene coding for the epidermal structure protein filaggrin allow elevated transepidermal water loss (TEWL), higher prevalence of *Staphylococcus aureus* (Staph Aureus) on the skin and facilitated penetration of allergens, e.g., from house dust mite (HDM). **(B)** Damaged keratinocytes (KC) release cytokines like interleukin-33 (IL-33), IL-25, and thymic stromal lymphopoietin (TSLP) which activate dermal ILC2. **(C)** Activated ILC2 produce high amounts of IL-13 which stimulates epidermal Langerhans cells (LC). LC migrate to regional lymph nodes to prime naïve T cells by antigen presentation via MHCII to promote development of T_H_2 cells that produce type II cytokines like IL-4, IL-5, and IL-13. **(D)** ILC2 can act as antigen presenting cells for T_H_2 effector cells through antigen presentation via MHCII and/or CD1a prompting them to produce IL-2 which in turn sustains ILC2 activation and survival. **(E)** ILC2 can be activated by mast cell (Mast) derived prostaglandin D_2_ (PGD_2_) and cysteinyl leukotrienes LTE_4_. ILC2 in turn produce IL-5 which promotes eosinophil (Eos) activation. Administration of montelukast can block LTE_4_-mediated activation of ILC2. IL-5 function can be therapeutically blocked by specific monoclonal antibodies like mepolizumab. MHCII, major histocompatibility complex II; TCR, T cell receptor.

Impaired barrier function of the skin is a hallmark in the pathogenesis of atopic dermatitis (AD). Loss-of-function-mutations in the gene coding for the epidermal structure protein filaggrin is strongly associated with an elevated risk to develop atopic dermatitis by allowing elevated trans epidermal water loss, higher prevalence of *Staphylococcus aureus* on the skin and facilitated penetration of allergens (58–61). The type 2 inflammatory response in AD is known to involve innate and adaptive immune cells like mast cells, eosinophils, and CD4^+^ T_H_2 cells, the latter producing type 2 cytokines like IL-4, IL-5, and IL-13 (62). Since ILC2 are described in the skin (63) this led to the hypothesis that innate lymphoid cells, especially ILC2, may contribute to the pathogenesis of this frequently occurring atopic disease (Figure 2).

### ILC in Human Atopic Dermatitis

Significantly more ILC2 can be found in lesional skin biopsies from patients suffering from atopic dermatitis in relation to skin from healthy individuals (25, 36). These ILC2 produce high amounts of the type 2 cytokines IL-5 and IL-13 and express the membrane bound IL-33 receptor ST2 as well-receptors for IL-25 and thymic stromal lymphopoietin (TSLP) (25, 36). These changes are even more profound when ILC2 are isolated from skin of house dust mite (HDM) allergic individuals that have been challenged epicutaneously with HDM extract. IL-33 is able to strongly enhance the expression of IL-13 and IL-5 and to increase the migratory capacity of isolated skin-derived ILC2 *in vitro* (36). Interestingly, ILC2 from atopic patients also express higher amounts of the killer cell lectin-like receptor G1 (KLRG1), which is even further elevated after stimulation with IL-33 or TSLP (36).

Human ILC2 express the prostaglandin D_2_ (PGD_2_) receptor chemoattractant receptor-homologous molecule expressed on T_H_2 cells (CRTH2) (64, 65). PGD_2_ which is mainly produced by mast cells induces ILC2 migration, production of type 2 cytokines and upregulation of the expression of IL-33 and IL-25 receptor subunits (ST2 and IL-17RA) *in vitro* (66). The effects of PGD_2_ on ILC2 can be mimicked by the supernatant from activated human mast cells (through IgE-mediated degranulation) and inhibited by a CRTH2 antagonist highlighting a cross-talk between mast cells and ILC2 (66).

ILC2 respond to further mast cell mediators like cysteinyl leukotrienes, particularly LTE_4_ (67). Human ILC express the functional leukotriene receptors CysLT_1_ and its expression is increased in patients with atopic dermatitis (67). LTE_4_ not only induces migration, promotes cytokine productions and upregulation of IL-33/IL-25 receptors in human ILC2 *in vitro*, but also enhances the pro-inflammatory effect of the epithelial cytokines IL-25, IL-33, TSLP, and of PGD2 as seen by increased production of IL-5 and IL-13. This effect of LTE_4_ can be partially inhibited by adding the leukotriene antagonist montelukast. Finally, addition of IL-2 to LTE_4_ and epithelial cytokines significantly further amplifies the activation of ILC2 (67). These findings clearly suggest a pathogenic role of ILC2 in the pathogenesis if atopic dermatitis in humans (Figure 2).

### ILC in Atopic Dermatitis Mouse Models

Topical application of a synthetic form of active vitamin D3 (MC903) to the skin of mice can mimic atopic dermatitis-like inflammation with a type 2 signature (68). Using the MC903 AD mouse model Salimi et al. and Kim et al. investigated inflammatory responses in the presence and absence of ILC2. When ILC2 are depleted in Rag1^−/−^ mice by administering an anti-CD90.2 and/or anti-CD25 monoclonal antibody this leads to an dramatically decreased ear swelling response (25, 36). Furthermore, using Rorα^sg/sg^ (Rorα-knockout) bone marrow chimeric mice which lack ILC2, a markedly reduced inflammatory response in the skin can be seen, highlighting ILC2 as a main proinflammatory cell in this type 2 inflammatory model (36). An increase in IL-33 and IL-25 expression has been reported in lesional skin of patients with AD compared with healthy individuals underlining an important role for these cytokines as proinflammatory ILC2 activating cytokines in AD (36, 69, 70). Strikingly, when flow cytometry assisted cell sorting (FACS)-purified ILC2 from MC903-treated C57BL/6 wild-type mice are adoptively transferred by intradermal injection into naïve C57BL/6 wild-type recipient mice, the recipient mice develop AD-like skin reactions with a type 2 T cell response indicating that these innate cells alone are capable of eliciting an AD-like skin response (25).

Another possible mouse model to study eczema like skin reactions are the “flaky tail” mice. These mice bear a frameshift mutation in the murine filaggrin gene (flg) resulting in expression of a truncated profilaggrin (~215 kDa) instead of the normal high-molecular-weight profilaggrin (>500 kDa) (71). Topical application of allergen to mice homozygous for this mutation results in cutaneous inflammatory infiltrates and enhanced cutaneous allergen priming with increased development of allergen-specific antibody responses (71). Saunders et al. characterized changes of ILC2 numbers and their cytokine production in flg-mutant mice (72). These mice show spontaneous atopic dermatitis-like inflammation and develop compromised pulmonary function. In the skin and skin draining lymph nodes of these mice, there is a significant increase in the frequency of IL-5-producing ILC compared to wild type animals. However, no differences in cell numbers are seen for ILC1 and 3. Furthermore, flg-mutant mice show higher skin infiltrates of eosinophils, mast cells and basophils (72). Even more astonishing, when flg-mutant mice are crossed with Rag1^−/−^ mice (Flg^ft/ft^Rag1^−/−^) skin lesions but not lung inflammation occur as shown by cutaneous expansion of IL-5-producing ILC2, indicating that skin inflammation can develop independently of the adaptive immune system in these mice (72). Regulation of ILC responses by adaptive immune cells is also reported in other tissues (73). Finally, increased frequency of ILC2 can be found in skin blisters taken from non-lesional skin of patients with filagrin mutations compared with the skin of filagrin wildtype subjects (72). Taken together, loss of filagrin function in humans and mice is clearly linked to increased ILC2 activation and disease progression in atopic dermatitis.

This latter model, however, has been challenged recently by the work of Schwartz et al. which provides evidence that atopic dermatitis like lesions can evolve independent of ILC2 and ILC2-derived cytokines in Filaggrin-mutant (Flg^ft/ft^) mice bred on an ILC2-deficient background (74). Interestingly, inflammation in these mice following MC903 treatment requires IL-1β and IL-1R1-signaling but is independent of NOD-, LRR- and pyrin domain-containing protein 3 (NLRP3) inflammasome activation and results in elevated numbers of IL-1β-responsive connective tissue mast cells (74). Finally, Flg^ft/ft^ mice do not develop skin inflammation under germ-free compared to SPF conditions indicting a crucial role for the microbiome in promoting proinflammatory immune responses in this mouse model (74). This issue will be discussed in more detail in a later section.

### ILC2 as Possible Therapeutic Targets in AD

Development of ILC2 depends on the transcription factor receptor-related orphan receptors alpha (RORα) and lack of RORα results in impaired lung inflammation in response to protease allergen in mice despite normal T_H_2 cell responses (75). Dai et al. provide evidence that a synthetic RORα/γ inverse agonist (SR1001) is able to suppress inflammation in the MC903-induced atopic dermatitis mouse models. Topical treatment with SR1001 reduces epidermal and dermal inflammation, suppresses the production of type 2 cytokines and TSLP, and reverses impaired keratinocyte differentiation (76). Since SR1001 also inhibits RORγ signaling it is quite possible that RORγt^+^ ILC3 functions may also be impaired (42). If topical inverse agonists for RORα may have anti-inflammatory functions in humans remains to be elucidated.

A crucial role for the IL-33/ILC2 axis in the pathogenesis of AD has been proposed by Imai et al. The authors generated a transgenic mouse line which overexpresses IL-33 in keratinocytes. These mice spontaneously develop an itchy dermatitis closely resembling AD at age 6–8 weeks with thickened epidermis, skin infiltration of eosinophils and mast cells, and high histamine and IgE levels in the blood (77). Moreover, IL-5 and IL-13 expressing ILC2 numbers are significantly increased in lesional skin, peripheral blood, and regional lymph nodes. Administering a neutralizing monoclonal anti-IL-5 antibody results in a marked reduction of the inflammatory response as shown by a decreased peripheral blood eosinophil count, milder thickened epidermis and lower inflammatory infiltrates including eosinophils (77). Unfortunately, a randomized, placebo-controlled parallel group design study in patients with AD could not detect a clinical improvement by administering a monoclonal antibody to human interleukin-5 (mepolizumab) in two single doses of 750 mg, given 1 week apart, despite a significant decrease in peripheral blood eosinophils (78).

## Role of ILC in Allergic Contact Dermatitis

**Figure 3:**
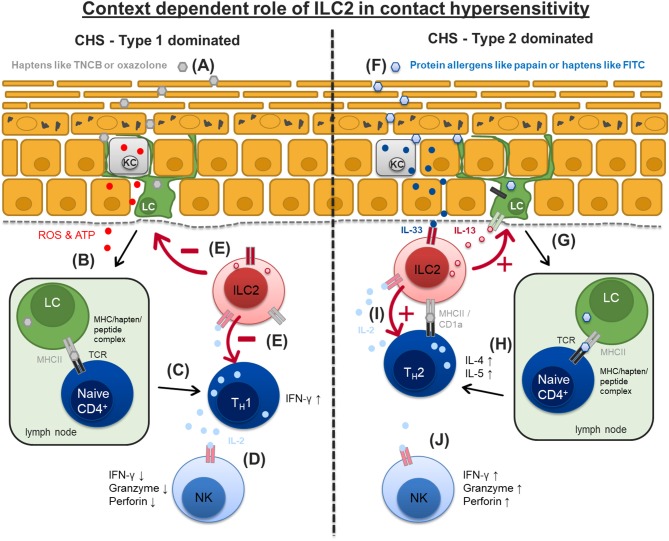
Context dependent role of ILC2 in type 1 and type 2 dominated contact hypersensitivity. Contact allergens irritate and penetrate the upper skin layers. **(A)** Typical contact allergens like TNCB or oxazololone cause ROS and ATP release from damaged keratinocytes (KC) and uptake by epidermal Langerhans cells (LC). **(B,C)** LC migrate to skin draining lymph nodes and promote a type 1 driven immune response mediated by T_H_1 and NK cells resulting in increased IFN-γ and IL-2 production. **(D)** Higher availability of IL-2 for NK cells results in their enhanced activation and effector cytokine production (IFN-γ, granzyme, perforin). **(E)** ILC2 are likely to suppress LC migration, T_H_1 polarization and NK cells activation in this type 1 CHS response via mechanisms that are currently not well-understood. **(F)** Protein allergens like papain or haptens like FITC cause IL-33 release from keratinocytes (KC) which in turn activates dermal ILC2 to produce large amounts of IL-13. **(G,H)** ILC2 derived IL-13 promotes trafficking of Langerhans cells (LC) to regional lymph nodes where they prime naïve T cells by interaction of MHCII/peptide/hapten complex and T cell receptor promoting the development of T_H_2 cells and a type 2 biased immune response. **(I)** ILC2 can act as antigen presenting cells for T_H_2 effector cells prompting them to produce IL-2 which in turn sustains ILC2 activation and survival. **(J)** ILC2 compete with other innate effector cells like NK cells for the survival factor IL-2 leading to a reduced/moderate NK cell activation. FITC, Fluorescein isothiocyanate; MHCII, major histocompatibility complex II; ROS, reactive oxygen species; TCR, T cell receptor; NK, natural killer cell; ATP, Adenosine triphosphate.

Allergic contact dermatitis (ACD) is a prevalent inflammatory skin disease triggered by low molecular weight organic chemicals or metal ions which penetrate the skin and bind covalently or by complex formation to proteins thereby activating the innate and adaptive immune response. ACD can be separated into two phases. The sensitization phase, were antigen upon first encounter with the skin is taken up by dendritic cells and transferred to the regional draining lymph nodes to be presented to antigen specific T-cells for priming. And the elicitation phase that is induced by subsequent antigen contact and leads to an infiltration of antigen-specific T-cells into the skin peaking 24–48 h after second antigen contact. In the mouse model of ACD, the contact hypersensitivity (CHS) model, hapten-specific CD8^+^ cytotoxic T-cells are thought to be the key effector cells in the elicitation phase rendering CHS a classical type 1 driven adaptive immune response (Figure 3). Typical haptens used in these models comprise oxazolone, 2,4,6-Trinitrochlorobenzene (TNCB) or 2,4-dinitrofluorobenzene (DNFB) (79–81). In addition, we and others have previously demonstrated that sensing of danger signals by cells of the innate immune system including dendritic cells, neutrophils, and mast cells represent a crucial element in the initiation and elicitation of CHS responses (82–86).

### NK Cells in Type 1 Dominated CHS Responses

Group 1 ILCs consisting of NK cells and ILC1 are involved in inflammatory bowel and allergic skin diseases in mice (12, 24, 87, 88). Regarding ACD Carbone et al. were able to characterize CD56^high^CD16^−^CD62L^−^ NK cells in an *ex vivo* human model which accumulate in affected skin of hapten allergic human individuals and these NK cells release type 1 cytokines and induce keratinocyte apoptosis *in vitro* (23). In mice NK cells can be further subdivided into two distinct subsets: CD49a^+^DX5^−^ liver-resident (Trail^+^) and CD49a^−^DX5^+^ conventional NK cells (cNK) (12). Furthermore, cNK cells seem to express much higher amounts of the transcription factor EOMES (87). Liver-resident NK cells can mediate long-lived, antigen-specific adaptive recall responses to haptens like DNFB and oxazolone independent of B cells and T cells (24). Preceding was the finding that a CHS response to several haptens can be elicited in Rag2^−/−^ mice lacking T- and B-cells but not in mice that either contain dysfunctional NK cells (SCID × beige mice) or completely lack NK cells (Rag2^−/−^ Il2rg^−/−^ mice). A proper CHS response can be transferred by FACS-purified antigen-specific Thy-1^+^ Ly49C-I^+^ liver-resident NK cells from sensitized Rag2^−/−^ mice when transferred into naive Rag2^−/−^ Il2rg^−/−^ recipients (24). The same NK cell type seems to mount antigen specific immunity against certain viral pathogens as well (88). Our own investigations using the hapten TNCB support the role of EOMES^+^ cNK cells as the dominant proinflammatory innate cell type in the early phase of contact hypersensitivity. NK cell numbers increase significantly 24 h in the ear skin of mice after allergen challenge and produce type 1 marker cytokines like IFN-γ and TNF (89). Taken together, NK cells seem to represent a major driving force of the innate immune system in CHS pathogenesis pathogenesis (Figures 3A–D).

### Helper-Like ILC in Type 1 Dominated CHS Responses

Very little is known about the involvement of helper-like ILC in the pathogenesis of CHS, however there has been some indirect evidence for it in the past. ILC2 are known to be a major source of IL-13 production thus playing a crucial role in innate type 2 immune responses to worms and inhaled allergens (90, 91). IL-13-deficient mice (Il13^−/−^) show impaired T_H_2 responses induced by epicutaneous ovalbumin (OVA) exposure whereas i.p. sensitization is normal and results in responses equivalent to wild type mice (92). Interestingly, Il13^−/−^ mice display an even enhanced ear swelling responses to the hapten DNFB, which is also known to elicit a type 1 T-cell driven immune response (93), compared to wild type mice. At the time, this finding was interpreted as a lack of T_H_2-mediated suppression but it's tempting to speculate that impaired ILC2 function in this mouse model may also have contributed to a disinhibited and thus exaggerated type 1 immune response. We recently characterized cell numbers and cytokine production of all ILC subgroups (ILC1-3 and NK cells) during the elicitation phase of a CHS mouse model based on the hapten TNCB using an ILC reporter system (89). Numbers of ILC are elevated in skin draining lymph nodes, show an activated phenotype and produce elevated amounts of their marker cytokines IL-13 and IL-5 at late time points (48 and 72 h), i.e., during the resolution phase of the inflammatory response in the skin. On the other hand, NK cell numbers and their production of IFN-γ and TNF are highest 24 h after allergen challenge paralleling the strongest skin inflammation period (89). The latter is expected since TNCB is known to elicit a type 1 driven immune response (93, 94). However, lack of ILC achieved by either antibody mediated depletion using an anti CD90.2 mAb in Rag1^−/−^ mice or by using mice that selectively lack ILC2 [Rorα^sg/flox^Il7r^Cre/+^mice (29)] results in a significantly enhanced and long lasting inflammatory response (89). The ear infiltrate of ILC depleted mice show a tendency toward a more type 1 biased immune response indicated by increased numbers of T-bet^+^ CD4^+^ T-cells (89). This data supports the concept of a counter regulatory role for ILC2 in CHS (Figures 3A–D).

### Helper-Like ILC in Type 2 Dominated Allergic Skin Responses

Some allergens like Fluorescein isothiocyanate (FITC) and papain rather induce allergic type 2 immune responses with increased IL-4 producing T_H_2 cell infiltrates in murine skin when reapplied topically or intradermally (28, 95, 96), suggesting that ILC2 might rather play a proinflammatory role in these models. Along this line we demonstrated in a papain skin challenge model that lack of IL-13-producing ILC2 leads to a marked reduction of inflammation with less skin infiltrating T_H_2 cells [(28); (Figures 3F–J)]. A first therapeutic approach in type 2 dominated allergic skin responses has been proposed by Bao et al. They demonstrate that ILC2 numbers are increased in the skin of FITC-challenged mice. In addition, intraperitoneal injection of the cycloartane triterpene saponin Astragaloside IV during the sensitization phase leads to a reduction of the inflammatory response as seen by a decreased ear swelling response, less production of pro-allergic cytokines like IL-33 and TSLP, and significantly reduced numbers of ILC2 in the skin of these mice (97). Thus, ILC2 seem to have contrary roles in type 1 and type 2 dominated allergic skin reactions, respectively (Figure 3).

## Role of Dermal ILC2 in Innate and Adaptive Immune Cross Talk

### Antigen Presentation by MHCII

ILC2 and ILC3 express MHCII molecules on their surface and can act as antigen presenting cells for helper T cells (29, 51, 52). Our own analysis of MHCII expression on ILC2 revealed that in skin draining lymph nodes of mice ~50% of the ILC2 express MHCII, while in the skin only ~3% express MHCII. Antibody mediated depletion of ILC leads to a significant reduction of MHCII positive ILC2 both in skin and LN (89). Currently, we can only speculate that ILC2 might regulate effector T cells in a direct fashion via MHCII. In line with this, Oliphant et al. recently demonstrated that MHCII expression on ILC2 and subsequent antigen presentation to CD4^+^ T cells is crucial for successful helminth expulsion in mice (29). The crosstalk between ILC2 and CD4^+^ T cells seems to involve IL-2 since activated CD4^+^ T cell-derived IL-2 has been shown to synergize with IL-33 to stimulate ILC2 (29). Thus, lack of ILC2 may lead to a higher availability of IL-2 for proliferation of other effector cells like NK cells leading to an augmented response in CHS.

### Antigen Presentation by CD1a

Another way how ILC2 might crosslink innate and adaptive immunity is by expressing the lipid-presenting molecule CD1a. Other than classical MHC proteins that present peptides, CD1 molecules present endogenous and exogenous lipid antigens to T lymphocytes (98). In a CHS model using the poison ivy-derived lipid contact allergen urushiol, CD1a expressing Langerhans cells are important to promote CD1a-restricted CD4^+^ T cells to produce IL-17 and IL-22. Furthermore, treatment with blocking antibodies against CD1a alleviates skin inflammation dramatically (99). More recently Hardman et al. demonstrated in a human skin challenge model that skin-derived ILC2 not only express CD1a but are also capable of helping CD1a-reactive T cells to sense *S. aureus* components in an cytosolic phospholipase A2 (PLA2G4A) and TLR-dependent–dependent manner, suggesting a new role for ILC2 in lipid surveillance of the skin (100). Currently, it is unclear whether this also applies for the adaptive immune response against urushiol. Taken together CD1a expression on ILC2 seems to be clearly involved in shaping the phenotype of adaptive T cell responses.

### Crosstalk With Basophils and Macrophages

Mashiko et al. reported significantly elevated frequencies of basophils, ILC and T_H_2 cells in the lesional skin of AD patients compared to patients suffering from psoriasis. Interestingly, basophils and ILC2 are positively correlated in skin, whereas skin basophils are inversely correlated with blood ILC2 suggesting that skin basophils may attract circulating ILC2 to skin of AD patients by IL-4 production (101). Kim et al. detected elevated numbers of basophils and ILC that form clusters in inflamed human AD skin compared to control skin. Using the MC903-based AD mouse model in IL-4/GFP reporter mice, they demonstrated that murine basophil responses preceded ILC2 responses and those basophils are the dominant IL-4-producing cell type in inflamed skin. In addition, ILC2 express the IL-4 receptor IL-4Rα and proliferate in an IL-4-dependent manner. Finally using Il4^−/−^ mice Kim et al. provide evidence that especially basophil-derived IL-4 is necessary for proinflammatory ILC2 responses in the skin (102).

Most notably, Egawa et al. have shown that basophil-derived IL-4 converts Ly6C^+^CCR2^+^ inflammatory monocytes into anti-inflammatory M2 macrophages in an IgE-mediated chronic allergic inflammation (IgE-CAI) mouse model, a model where basophils rather than mast cells and T cells play a critical role for the elicitation of allergic response (103, 104). In this model, skin infiltrating monocytes acquire an M2-like phenotype in an IL-4R- and basophil-dependent manner and adoptive transfer of Ly6C^+^CCR2^+^ inflammatory monocytes dampens the exacerbated IgE-CAI in CCR2^−/−^ mice which also requires IL-4R signaling (103). Thus, it is tempting to speculate, that basophil-derived IL-4 may promote pro-inflammatory responses via ILC2 and anti-inflammatory signals via M2 macrophages at the same time, leading to a counterbalanced immune response. However, the role of ILC2 in the IgE-CAI model is not known so far.

On the other side, ILC2 have been shown to promote polarization of the anti-inflammatory M2 macrophages by producing type-2 cytokines (IL-4, IL-5, and IL-13) in an renal ischemia-reperfusion injury model and experimental cerebral malaria (105, 106). Furthermore, in obese mice PD-1^high^ ILC2 are inhibited by PD-L1 expressing M1 macrophages which is promoted by TNF. PD-1 blockade improves ILC2 function, reinforces type 2 innate responses and promotes adipose tissue homeostasis (107, 108). Interestingly, in an serum-induced arthritis mouse model ILC2 were indispensable for dampening proinflammatory IL-1β secretion by bone marrow-derived macrophages (109). Finally, basophil-derived IL-4 seems to be essential for M2 macrophage mediated trapping of Nippostrongylus Brasiliensis larvae in the skin during second infection of mice thereby leading to reduced worm burden in the lung (110). However, basophils had no apparent contribution to worm expulsion from the intestine highlighting their crucial role in the skin (110).

Taken together, there seems to be an intense crosstalk between basophils, ILC2 and macrophages involving cytokines like IL-4, IL-13, and IL1β and resulting in differential polarization of macrophages dependent on the disease model. How these three cell type interact in AD and CHS remains to be elucidated.

### Crosstalk With Dendritic Cells

Using the protease-allergen papain which induces type 2 allergic airway and skin inflammation we showed that ILC2 are necessary for mounting an appropriate antigen specific T_H_2 memory response and that ILC2 activation clearly precedes T_H_2 involvement in papain induced airway and skin inflammation (28). Furthermore, ILC2-derived IL-13 is needed for the activation and expansion of an allergen-induced subset of dendritic cells (CD11b^+^CD103^−^IRF4^+^) which produce the T_H_2 cell chemoattractant CCL17. Using ILC2-deficient mice, we demonstrated that dermal ILC2 are crucial to mediate expansion of CCL17^+^ dendritic cells after skin challenge with papain finally leading to an effective T_H_2 memory response. Thus, ILC2 licensing of dendritic cells is a critical component of the memory T_H_2 cell response to certain allergens at barrier sites (28).

## Influence of Skin Microbiota on ILC2 Immunity

As mentioned earlier, filaggrin mutant mice significantly differ in their microbiome composition compared to wild type mice and do not develop skin inflammation under germ-free conditions prompting a crucial role for the microbiome in shaping this setting (74). Several studies have investigated the role of skin commensal bacteria in shaping the host immune cell functions of this organ (111–113). This mostly involves skin derived dendritic cells as sensors of bacterial antigens which promote development of commensal-specific T cells. These T cells help to improve tissue repair and protection to pathogens rendering them as important players in the skin homeostasis (111, 112).

When analyzing different skin-derived bacterial strains in a pediatric AD cohort over time, Byrd et al. were able to detect certain clonal *S. aureus* strains which are associated with more severe disease (113). Interestingly, heterogeneous *Staphylococcus epidermidis* strains were found in patients with less severe disease indicating that clonal expansion of certain bacterial strains can trigger proinflammatory responses in human AD. Furthermore, *S. aureus* isolates from AD patients with more severe flares can induce epidermal thickening and expansion of cutaneous T_H_2 and T_H_17 cells in a murine AD model (113).

These findings are suggesting a role of the microbiome to shape ILC2 functions as well. Interestingly, ILC2 distribution and homeostatic function in bone marrow, fat, lung, gut, and skin seems to be independent of commensal microbiota when comparing SPF to germ free mice (114). However, in mouse model of chronic obstructive pulmonary disease (COPD), challenge with *S. aureus* or *Haemophilis influenzae* lead to loss of GATA-3 expression in ILC2 and a subsequent increase in the expression of IL-12Rβ2, IL-18Rα, and T-bet giving them an ILC1-like phenotype (115). This ILC2 plasticity can also be influenced by viral stimuli especially influenza A virus (115).

Taken together, there is substantial evidence that the microbiome is involved in shaping ILC2 function and plasticity, especially in inflammatory lung diseases. Whether this concept also applies to the pathogenesis of inflammatory skin disease like AD and CHS remains to be determined.

## Type 1 and Type 2 Counter Regulation in CHS

Type 1 and type 2 immune responses are known to tightly counter-regulate each other (116). T_H_1 cytokines such as IFN-γ have been shown to antagonize the function of ILC2 and type 2 innate immune responses in mouse models of allergic lung inflammation and viral respiratory tract infections (13, 117). ILC2-mediated lung inflammation is enhanced in the absence of the IFN-γ receptor on ILC2 cells *in vivo* and IFN-γ effectively suppresses the function of tissue-resident ILC2 cells, two observations that clearly suggest a suppressive function of type 1 cytokines on ILC2 (13). Our own investigations reveal that TNCB based CHS in a mouse model is counter regulated by activated ILC, since lack of all ILC or ILC2 alone leads to a dramatic increase in the inflammatory response with a type 1 immune response bias (89). More recently, it has been reported that in the early stage of papain-induced lung inflammation in mice, depletion of NK cells results in increased numbers and cytokine production of ILC2, suggesting that NK cells negatively regulate ILC2 (118). Hapten based CHS experiments in Il15^−/−^ mice, which lack NK cells, demonstrate dramatically reduced ear swelling responses and at the same time increased numbers of ILC2 in skin and skin draining lymph nodes (89). Thus, a mutual balance between type 1 and type 2 immunity may also exist in CHS, in which NK cells negatively regulate ILC2 and ILC2 counter regulate type 1 immune responses mainly driven by NK cells, T_H_1, and T_C_1 cells.

Recently, Kim et al. identified IL-10-producing lineage negative lymphoid cells that show elevated numbers in the axillary as well as inguinal lymph nodes and ear tissues of Oxazolone challenged mice suggesting a possible regulatory role of ILC (119). These cells were designated “ILC10” and identified by expression of markers like CD45, CD127, and Sca-1, while detailed characterization of the exact ILC subpopulation was not provided. Along the same line, an IL-10 producing ILC2 effector cell population has recently been described in murine lung and suggested to regulate immune responses in a papain induced allergic lung inflammation model (4). These studies prompted us to address the presence of IL-10 producing ILC. Using highly sensitive IL-10 transcriptional reporter mice (120) we, however, could not identify relevant numbers of IL-10 transcribing lineage negative cells in different tissues (skin, lymph nodes, blood, and spleen) in the TNCB induced CHS model (89). Thus, at least in our hands ILC derived IL-10 does not appear to be responsible for the regulatory effects of ILC in type 1 dominated CHS of the skin.

Nevertheless, ILC2 are reported to promote regulatory T (Treg) cell expansion, thus framing the hypothesis that ILC2 can regulate inflammation indirectly. Molofski et al. demonstrated that ICOSL expression by ILC2 can stimulate ICOS^+^ Treg cells, providing a potential indirect link between IL-33 and Treg cells (121). In line with this, Rauber et al. could demonstrate that IL-9 producing ILC2 are crucial in promoting Treg driven anti-inflammatory effects in an antigen-induced arthritis mouse model. This ILC2/Treg interaction was dependent on direct cell contact involving ICOS–ICOSL interaction (122).

We recently showed that IL-33-induced OX40L expression by ILC2 is critical for tissue-specific expansion of Treg cells (32). Moreover, our data indicates that OX40L/OX40-driven interactions between ILC2 and Treg cells preferentially expands GATA3^+^ Treg cells, which are thought to be tissue-resident and functionally primed (123). IL-33-induced OX40L expression by ILC2 and the associated Treg cell expansion seems to be restricted to specific anatomical locations such as the airway and adipose tissue but not LN or gut (32). Thus, it remains unknown if a similar mechanism or alternative ILC2-independent suppressive pathways are involved in the skin.

Malhotra et al. recently found skin resident RORα-expressing Tregs to dampen ILC2-driven inflammation in a mouse model for atopic dermatitis (124). This effect is thought to be based on the enhanced expression of TNF ligand–related molecule 1 (TL1A) and death receptor 3 (DR3) on ILC2 as well as suppressed IL-4 expression. RORα-expressing Tregs are found in higher numbers in human skin compared to peripheral blood suggesting a possible counter regulatory role for these cells in ILC2-driven allergic skin diseases (124).

Taken together, these data show that ILC2 can act as modulators of the adaptive immune response and that the functional outcome very much depends on the context of the inflammatory reaction that is analyzed. In type 2 dominated skin inflammation ILC2 seem to be primarily proinflammatory while in the context of a type 1 dominated immune response ILC2 can act as regulators that help to counterbalance the inflammatory reaction (Figure 3).

## Concluding Remarks and Outlook

Innate lymphoid cells are increasingly emerging as important effectors of the innate immune system finally shaping a distinctive adaptive immune response. This includes on the one side important physiological functions in promoting wound healing, adipose tissue homeostasis, protection from pathogens and dampening of certain inflammatory disorders via Treg induction. On the other side, ILC2 have been shown to be important proinflammatory players in diseases like allergic asthma and atopic dermatitis. In the case of atopic dermatitis ILC2 have been described to be the major proinflammatory ILC subtype accountable for the production of marker cytokines like IL-13 and IL-5, cross-talk with other innate cells like basophils and dendritic cells, and finally promoting the development of T_H_2 cells. ILC2 will continue to be of high interest as possible targets in AD therapy, especially concerning their potential to produce high amounts of cytokines.

Immunologic reaction in allergic contact dermatitis can differ depending on the type of hapten used. Haptens like TNCB or oxazolone inducing type 1 responses clearly favor NK cells and T_H_1 cells as the driving proinflammatory force. In these models ILC2 may have counter regulatory functions as our own investigations suggest. On the other side, in allergic type 2 responses of the skin, induced by distinct haptens like FITC or protein allergens like papain, ILC2 seem to have a proinflammatory role. These observations clearly emphasize a context dependent function of ILC2 which is determined by the type of model analyzed (type 1 or type 2 dominated).

Additionally, ILC2 have recently been shown to be part of a neuro-immune interface. ILC2 function can be influenced by the neuropeptide neuromedin U (NMU) secreted by cholinergic neurons in the mucosal tissue of the gut and lungs. This goes in line with other studies showing that further neuroendocrine factors like norepinephrine, vasoactive intestinal peptide (VIP), calcitonin gene-related peptide (CGRP), and acetylcholine can modify ILC2 function as well (125–131). Furthermore, challenge of mouse skin with the poison ivy compound urushiol leads to an increase in IL-33 expression which can act on small to medium-sized dorsal root ganglion neurons that innervate the skin and express the IL-33 receptor ST2 (132). Strikingly, targeting IL-33 by either neutralizing antibodies or intrathecal application of ST2 siRNA results in significantly reduced itching and subsequently less scratching behavior in these mice, suggesting a new therapeutic approach in poison ivy ACD (132). Since pruritus is a hallmark symptom of ACD in humans and mice which is mediated by certain sensory neurons (133) it is tempting to speculate that this new identified “neuron-ILC2 unit” may also be important in the pathogenesis of AD and ACD. This hypothesis is further supported by studies showing that type 2 cytokines like TSLP and IL-4 can enhance itching (134, 135).

Taken together, the picture of ILC function in allergic skin diseases is far from complete. Further investigations especially on the mode of action of how ILC modify immune responses in a context dependent fashion are needed to fill this gap of knowledge.

## Author Contributions

DR-S did the main research and wrote the first draft of the manuscript. CK, TH, and YT provided substantial contributions to acquisition, analysis, and interpretation of the scientific content of this work. TJ provided the main contribution to the conception and design of the work. All authors contributed to manuscript revision, read, and approved the submitted version.

### Conflict of Interest

The authors declare that the research was conducted in the absence of any commercial or financial relationships that could be construed as a potential conflict of interest.

## References

[1] Constantinides MG McDonald BD Verhoef PA Bendelac A A committed hemopoietic precursor to innate lymphoid cells. Nature. (2014) 508:397–401. 10.1038/nature13047 24509713 PMC4003507

[2] Eberl G Colonna M Santo JPD McKenzie ANJ. Innate lymphoid cells: a new paradigm in immunology. Science. (2015) 348:aaa6566. 10.1126/science.aaa6566 25999512 PMC5658207

[3] Klose CS Diefenbach A. Transcription factors controlling innate lymphoid cell fate decisions. Curr Top Microbiol Immunol. (2014) 381:215–55. 10.1007/82_2014_381 25038936

[4] Seehus CR Kadavallore A Torre B de la Yeckes AR Wang Y Tang J. Alternative activation generates IL-10 producing type 2 innate lymphoid cells. Nat Commun. (2017) 8:1900. 10.1038/s41467-017-02023-z 29196657 PMC5711851

[5] Crome SQ Nguyen LT Lopez-Verges S Yang SYC Martin B Yam JY. A distinct innate lymphoid cell population regulates tumor-associated T cells. Nat Med. (2017) 23:368–75. 10.1038/nm.4278 28165478 PMC5497996

[6] Wang S Xia P Chen Y Qu Y Xiong Z Ye B. Regulatory innate lymphoid cells control innate intestinal inflammation. Cell. (2017) 171:201–16.e18. 10.1016/j.cell.2017.07.027 28844693

[7] Klose CSN Artis D. Innate lymphoid cells as regulators of immunity, inflammation and tissue homeostasis. Nat Immunol. (2016) 17:765–74. 10.1038/ni.3489 27328006

[8] Vosshenrich CAJ García-Ojeda ME Samson-Villéger SI Pasqualetto V Enault L Goff OR-L. A thymic pathway of mouse natural killer cell development characterized by expression of GATA-3 and CD127. Nat Immunol. (2006) 7:1217–24. 10.1038/ni1395 17013389

[9] Veinotte LL Halim TYF Takei F. Unique subset of natural killer cells develops from progenitors in lymph node. Blood. (2008) 111:4201–8. 10.1182/blood-2007-04-087577 18227350

[10] Huang Y Mao K Chen X Sun M Kawabe T Li W. S1P-dependent interorgan trafficking of group 2 innate lymphoid cells supports host defense. Science. (2018) 359:114–9. 10.1126/science.aam5809 29302015 PMC6956613

[11] Gasteiger G Fan X Dikiy S Lee SY Rudensky AY Tissue residency of innate lymphoid cells in lymphoid and non-lymphoid organs. Science. (2015) 350:981–5. 10.1126/science.aac9593 26472762 PMC4720139

[12] Peng H Jiang X Chen Y Sojka DK Wei H Gao X. Liver-resident NK cells confer adaptive immunity in skin-contact inflammation. J Clin Invest. (2013) 123:1444–56. 10.1172/JCI66381 23524967 PMC3613925

[13] Moro K Kabata H Tanabe M Koga S Takeno N Mochizuki M. Interferon and IL-27 antagonize the function of group 2 innate lymphoid cells and type 2 innate immune responses. Nat Immunol. (2016) 17:76–86. 10.1038/ni.3309 26595888

[14] Bando JK Liang H-E Locksley RM. Identification and distribution of developing innate lymphoid cells in the fetal mouse intestine. Nat Immunol. (2015) 16:153–60. 10.1038/ni.3057 25501629 PMC4297560

[15] Schuijs MJ Halim TYF. Group 2 innate lymphocytes at the interface between innate and adaptive immunity. Ann N Y Acad Sci. (2018) 1417:87–103. 10.1111/nyas.13604 29492980

[16] Klose CSN Blatz K d'Hargues Y Hernandez PP Kofoed-Nielsen M Ripka JF. The transcription factor T-bet is induced by IL-15 and thymic agonist selection and controls CD8^αα+^ intraepithelial lymphocyte development. Immunity. (2014) 41:230–43. 10.1016/j.immuni.2014.06.018 25148024

[17] Gordon SM Chaix J Rupp LJ Wu J Madera S Sun JC. The transcription factors T-bet and Eomes control key checkpoints of natural killer cell maturation. Immunity. (2012) 36:55–67. 10.1016/j.immuni.2011.11.016 22261438 PMC3381976

[18] Sojka DK Plougastel-Douglas B Yang L Pak-Wittel MA Artyomov MN Ivanova Y. Tissue-resident natural killer (NK) cells are cell lineages distinct from thymic and conventional splenic NK cells. eLife. (2014) 3:e1659. 10.7554/eLife.01659 24714492 PMC3975579

[19] Daussy C Faure F Mayol K Viel S Gasteiger G Charrier E. T-bet and Eomes instruct the development of two distinct natural killer cell lineages in the liver and in the bone marrow. J Exp Med. (2014) 211:563–77. 10.1084/jem.20131560 24516120 PMC3949572

[20] Abt MC Lewis BB Caballero S Xiong H Carter RA Sušac B. Innate immune defenses mediated by two ILC subsets are critical for protection against acute clostridium difficile infection. Cell Host Microbe. (2015) 18:27–37. 10.1016/j.chom.2015.06.011 26159718 PMC4537644

[21] Spits H Bernink JH Lanier L. NK cells and type 1 innate lymphoid cells: partners in host defense. Nat Immunol. (2016) 17:758–64. 10.1038/ni.3482 27328005

[22] Fuchs A Vermi W Lee JS Lonardi S Gilfillan S Newberry RD. Intraepithelial type 1 innate lymphoid cells are a unique subset of IL-12- and IL-15-responsive IFN-gamma-producing cells. Immunity. (2013) 38:769–81. 10.1016/j.immuni.2013.02.010 23453631 PMC3634355

[23] Carbone T Nasorri F Pennino D Eyerich K Foerster S Cifaldi L. CD56^high^CD16^−^CD62L^−^ NK cells accumulate in allergic contact dermatitis and contribute to the expression of allergic responses. J Immunol. (2010) 184:1102–10. 10.4049/jimmunol.0902518 20008290

[24] O'Leary JG Goodarzi M Drayton DL von Andrian UH. T cell- and B cell-independent adaptive immunity mediated by natural killer cells. Nat Immunol. (2006) 7:507–16. 10.1038/ni1332 16617337

[25] Kim BS Siracusa MC Saenz SA Noti M Monticelli LA Sonnenberg GF. TSLP elicits IL-33–independent innate lymphoid cell responses to promote skin inflammation. Sci Transl Med. (2013) 5:170ra16. 10.1126/scitranslmed.3005374 23363980 PMC3637661

[26] Rak GD Osborne LC Siracusa MC Kim BS Wang K Bayat A. IL-33-dependent group 2 innate lymphoid cells promote cutaneous wound healing. J Invest Dermatol. (2015) 136:487–96. 10.1038/JID.2015.406 26802241 PMC4731037

[27] Christianson CA Goplen NP Zafar I Irvin C Good JT Jr Rollins DR. Persistence of asthma requires multiple feedback circuits involving type 2 innate lymphoid cells and IL-33. J Allergy Clin Immunol. (2015) 136:59–68.e14. 10.1016/j.jaci.2014.11.037 25617223 PMC4494983

[28] Halim TY Hwang YY Scanlon ST Zaghouani H Garbi N Fallon PG Group 2 innate lymphoid cells license dendritic cells to potentiate memory T helper 2 cell responses. Nat Immunol. (2016) 17:57–64. 10.1038/ni.3294 26523868 PMC4685755

[29] Oliphant CJ Hwang YY Walker JA Salimi M Wong SH Brewer JM. MHCII-mediated dialog between group 2 innate lymphoid cells and CD4^+^ T cells potentiates type 2 immunity and promotes parasitic helminth expulsion. Immunity. (2014) 41:283–95. 10.1016/j.immuni.2014.06.016 25088770 PMC4148706

[30] Paclik D Stehle C Lahmann A Hutloff A Romagnani C. ICOS regulates the pool of group 2 innate lymphoid cells under homeostatic and inflammatory conditions in mice. Eur J Immunol. (2015) 45:2766–72. 10.1002/eji.201545635 26249010

[31] Maazi H Patel N Sankaranarayanan I Suzuki Y Rigas D Soroosh P. ICOS:ICOS-ligand interaction is required for type 2 innate lymphoid cell function, homeostasis, and induction of airway hyperreactivity. Immunity. (2015) 42:538–51. 10.1016/j.immuni.2015.02.007 25769613 PMC4366271

[32] Halim TYF Rana BMJ Walker JA Kerscher B Knolle MD Jolin HE. Tissue-restricted adaptive type 2 immunity is orchestrated by expression of the costimulatory molecule OX40L on group 2 innate lymphoid cells. Immunity. (2018) 48:1195–207.e6. 10.1016/j.immuni.2018.05.003 29907525 PMC6015114

[33] Smith SG Chen R Kjarsgaard M Huang C Oliveria J-P O'Byrne PM. Increased numbers of activated group 2 innate lymphoid cells in the airways of patients with severe asthma and persistent airway eosinophilia. J Allergy Clin Immunol. (2016) 137:75–86.e8. 10.1016/j.jaci.2015.05.037 26194544

[34] Bartemes K Kephart G Fox SJ Kita H. Enhanced innate type 2 immune response in peripheral blood from patients with asthma. J Allergy Clin Immunol. (2014) 134:671–8.e4. 10.1016/j.jaci.2014.06.024 25171868 PMC4149890

[35] Liu T Wu J Zhao J Wang J Zhang Y Liu L. Type 2 innate lymphoid cells: a novel biomarker of eosinophilic airway inflammation in patients with mild to moderate asthma. Respir Med. (2015) 109:1391–6. 10.1016/j.rmed.2015.09.016 26459159

[36] Salimi M Barlow JL Saunders SP Xue L Gutowska-Owsiak D Wang X. A role for IL-25 and IL-33-driven type-2 innate lymphoid cells in atopic dermatitis. J Exp Med. (2013) 210:2939–50. 10.1084/jem.20130351 24323357 PMC3865470

[37] Gladiator A Wangler N Trautwein-Weidner K LeibundGut-Landmann S. Cutting edge: IL-17–secreting innate lymphoid cells are essential for host defense against fungal infection. J Immunol. (2013) 190:521–5. 10.4049/jimmunol.1202924 23255360

[38] Goto Y Obata T Kunisawa J Sato S Ivanov II Lamichhane A. Innate lymphoid cells regulate intestinal epithelial cell glycosylation. Science. (2014) 345:1254009. 10.1126/science.1254009 25214634 PMC4774895

[39] Hernández PP Mahlakoiv T Yang I Schwierzeck V Nguyen N Guendel F Interferon-λ and interleukin-22 cooperate for the induction of interferon-stimulated genes and control of rotavirus infection. Nat Immunol. (2015) 16:698–707. 10.1038/ni.3180 26006013 PMC4589158

[40] Sonnenberg GF Monticelli LA Elloso MM Fouser LA Artis D. CD4^+^ lymphoid tissue inducer cells promote innate immunity in the gut. Immunity. (2011) 34:122–34. 10.1016/j.immuni.2010.12.009 21194981 PMC3035987

[41] Zheng Y Valdez PA Danilenko DM Hu Y Sa SM Gong Q. Interleukin-22 mediates early host defense against attaching and effacing bacterial pathogens. Nat Med. (2008) 14:282–9. 10.1038/nm1720 18264109

[42] Eberl G Marmon S Sunshine M-J Rennert PD Choi Y Littman DR An essential function for the nuclear receptor RORγt in the generation of fetal lymphoid tissue inducer cells. Nat Immunol. (2004) 5:64–73. 10.1038/ni1022 14691482

[43] Yokota Y Mansouri A Mori S Sugawara S Adachi S Nishikawa S-I. Development of peripheral lymphoid organs and natural killer cells depends on the helix–loop–helix inhibitor Id2. Nature. (1999) 397:702–6. 10.1038/17812 10067894

[44] Mebius RE Rennert P Weissman IL Developing lymph nodes collect CD4^+^CD3^−^ LTβ^+^ cells that can differentiate to APC, NK cells, and follicular cells but not T or B cells. Immunity. (1997) 7:493–504. 10.1016/S1074-7613(00)80371-4 9354470

[45] Sun Z Unutmaz D Zou Y-R Sunshine MJ Pierani A Brenner-Morton S. Requirement for RORγ in thymocyte survival and lymphoid organ development. Science. (2000) 288:2369–73. 10.1126/science.288.5475.2369 10875923

[46] Klose CSN Kiss EA Schwierzeck V Ebert K Hoyler T d'Hargues Y. A T-bet gradient controls the fate and function of CCR6-RORγt+ innate lymphoid cells. Nature. (2013) 494:261–5. 10.1038/nature11813 23334414

[47] Sciumé G Hirahara K Takahashi H Laurence A Villarino AV Singleton KL. Distinct requirements for T-bet in gut innate lymphoid cells. J Exp Med. (2012) 209:2331–8. 10.1084/jem.20122097 23209316 PMC3526352

[48] Vonarbourg C Mortha A Bui VL Hernandez PP Kiss EA Hoyler T. Regulated expression of nuclear receptor RORγt confers distinct functional fates to NK cell receptor-expressing RORγt+ innate lymphocytes. Immunity. (2010) 33:736–51. 10.1016/j.immuni.2010.10.017 21093318 PMC3042726

[49] Rankin L Groom JR Chopin M Herold M Walker JA Mielke LA T-bet is essential for NKp46+ innate lymphocyte development through the Notch pathway. Nat Immunol. (2013) 14:389–95. 10.1038/ni.2545 23455676 PMC4076532

[50] Bernink JH Peters CP Munneke M te Velde AA Meijer SL Weijer K. Human type 1 innate lymphoid cells accumulate in inflamed mucosal tissues. Nat Immunol. (2013) 14:221–9. 10.1038/ni.2534 23334791

[51] Hepworth MR Fung TC Masur SH Kelsen JR McConnell FM Dubrot J. Group 3 innate lymphoid cells mediate intestinal selection of commensal bacteria-specific CD4^+^ T cells. Science. (2015) 348:1031–5. 10.1126/science.aaa4812 25908663 PMC4449822

[52] Hepworth MR Monticelli LA Fung TC Ziegler CGK Grunberg S Sinha R. Innate lymphoid cells regulate CD4^+^ T-cell responses to intestinal commensal bacteria. Nature. (2013) 498:113–7. 10.1038/nature12240 23698371 PMC3699860

[53] Sonnenberg GF Monticelli LA Alenghat T Fung TC Hutnick NA Kunisawa J. Innate lymphoid cells promote anatomical containment of lymphoid-resident commensal bacteria. Science. (2012) 336:1321–5. 10.1126/science.1222551 22674331 PMC3659421

[54] Pantelyushin S Haak S Ingold B Kulig P Heppner FL Navarini AA. Rorgammat+ innate lymphocytes and gammadelta T cells initiate psoriasiform plaque formation in mice. J Clin Invest. (2012) 122:2252–6. 10.1172/JCI61862 22546855 PMC3366412

[55] Brüggen M-C Bauer WM Reininger B Clim E Captarencu C Steiner GE. *In situ* mapping of innate lymphoid cells in human skin: evidence for remarkable differences between normal and inflamed skin. J Invest Dermatol. (2016) 136:2396–405. 10.1016/j.jid.2016.07.017 27456756

[56] Teunissen MBM Munneke JM Bernink JH Spuls PI Res PCM te Velde A. Composition of innate lymphoid cell subsets in the human skin: enrichment of NCR+ ILC3 in lesional skin and blood of psoriasis patients. J Invest Dermatol. (2014) 134:2351–60. 10.1038/jid.2014.146 24658504

[57] Villanova F Flutter B Tosi I Grys K Sreeneebus H Perera GK. Characterization of innate lymphoid cells in human skin and blood demonstrates increase of NKp44+ ILC3 in psoriasis. J Invest Dermatol. (2014) 134:984–91. 10.1038/jid.2013.477 24352038 PMC3961476

[58] Irvine AD McLean WHI Leung DYM. Filaggrin mutations associated with skin and allergic diseases. N Engl J Med. (2011) 365:1315–27. 10.1056/NEJMra1011040 21991953

[59] Palmer CNA Irvine AD Terron-Kwiatkowski A Zhao Y Liao H Lee SP. Common loss-of-function variants of the epidermal barrier protein filaggrin are a major predisposing factor for atopic dermatitis. Nat Genet. (2006) 38:441–6. 10.1038/ng1767 16550169

[60] Rodríguez E Baurecht H Herberich E Wagenpfeil S Brown SJ Cordell HJ. Meta-analysis of filaggrin polymorphisms in eczema and asthma: robust risk factors in atopic disease. J Allergy Clin Immunol. (2009) 123:1361–70.e7. 10.1016/j.jaci.2009.03.036 19501237

[61] Sandilands A Terron-Kwiatkowski A Hull PR O'Regan GM Clayton TH Watson RM. Comprehensive analysis of the gene encoding filaggrin uncovers prevalent and rare mutations in ichthyosis vulgaris and atopic eczema. Nat Genet. (2007) 39:650–4. 10.1038/ng2020 17417636

[62] Scheerer C Eyerich K Pathogenese des atopischen Ekzems. Hautarzt. (2018) 69:191–6. 10.1007/s00105-018-4127-4 29404622

[63] Roediger B Kyle R Yip KH Sumaria N Guy TV Kim BS. Cutaneous immunosurveillance and regulation of inflammation by group 2 innate lymphoid cells. Nat Immunol. (2013) 14:564–73. 10.1038/ni.2584 23603794 PMC4282745

[64] Mjösberg JM Trifari S Crellin NK Peters CP van Drunen CM Piet B. Human IL-25- and IL-33-responsive type 2 innate lymphoid cells are defined by expression of CRTH2 and CD161. Nat Immunol. (2011) 12:1055–62. 10.1038/ni.2104 21909091

[65] Tait Wojno E Monticelli L Tran S Alenghat T Osborne L Thome J The prostaglandin D2 receptor CRTH2 regulates accumulation of group 2 innate lymphoid cells in the inflamed lung. Mucosal Immunol. (2015) 8:1313–23. 10.1038/mi.2015.21 25850654 PMC4598246

[66] Xue L Salimi M Panse I Mjosberg JM McKenzie ANJ Spits H. Prostaglandin D2 activates group 2 innate lymphoid cells through chemoattractant receptor-homologous molecule expressed on TH2 cells. J Allergy Clin Immunol. (2014) 133:1184–94.e7. 10.1016/j.jaci.2013.10.056 24388011 PMC3979107

[67] Salimi M Stöger L Liu W Go S Pavord I Klenerman P. Cysteinyl leukotriene E4 activates human group 2 innate lymphoid cells and enhances the effect of prostaglandin D2 and epithelial cytokines. J Allergy Clin Immunol. (2017) 140:1090–100.e11. 10.1016/j.jaci.2016.12.958 28115217 PMC5624780

[68] Li M Hener P Zhang Z Kato S Metzger D Chambon P. Topical vitamin D3 and low-calcemic analogs induce thymic stromal lymphopoietin in mouse keratinocytes and trigger an atopic dermatitis. Proc Natl Acad Sci USA. (2006) 103:11736–41. 10.1073/pnas.0604575103 16880407 PMC1544239

[69] Hvid M Vestergaard C Kemp K Christensen GB Deleuran B Deleuran M. IL-25 in Atopic Dermatitis: a Possible Link between Inflammation and Skin Barrier Dysfunction? J Invest Dermatol. (2011) 131:150–7. 10.1038/jid.2010.277 20861853

[70] Wang Y-H Angkasekwinai P Lu N Voo KS Arima K Hanabuchi S. IL-25 augments type 2 immune responses by enhancing the expansion and functions of TSLP-DC–activated Th2 memory cells. J Exp Med. (2007) 204:1837–47. 10.1084/jem.20070406 17635955 PMC2118667

[71] Fallon PG Sasaki T Sandilands A Campbell LE Saunders SP Mangan NE A homozygous frameshift mutation in the murine filaggrin gene facilitates enhanced percutaneous allergen priming. Nat Genet. (2009) 41:602–8. 10.1038/ng.358 19349982 PMC2872154

[72] Saunders SP Moran T Floudas A Wurlod F Kaszlikowska A Salimi M. Spontaneous atopic dermatitis is mediated by innate immunity, with the secondary lung inflammation of the atopic march requiring adaptive immunity. J Allergy Clin Immunol. (2016) 137:482–91. 10.1016/j.jaci.2015.06.045 26299987 PMC4735016

[73] Gasteiger G Rudensky AY Opinion: interactions of innate and adaptive lymphocytes. Nat Rev Immunol. (2014) 14:631–9. 10.1038/nri3726 25132095 PMC4504695

[74] Schwartz C Moran T Saunders SP Kaszlikowska A Floudas A Bom J. Spontaneous atopic dermatitis in mice with a defective skin barrier is independent of ILC2 and mediated by IL-1β. Allergy. (2019) 74:1920–33. 10.1111/all.13801 30937919 PMC6850072

[75] Halim TY MacLaren A Romanish MT Gold MJ McNagny KM Takei F. Retinoic-acid-receptor-related orphan nuclear receptor alpha is required for natural helper cell development and allergic inflammation. Immunity. (2012) 37:463–74. 10.1016/j.immuni.2012.06.012 22981535

[76] Dai J Choo M-K Park JM Fisher DE. Topical ROR inverse agonists suppress inflammation in mouse models of atopic dermatitis and acute irritant dermatitis. J Invest Dermatol. (2017) 137:2523–31. 10.1016/j.jid.2017.07.819 28774591 PMC5990371

[77] Imai Y Yasuda K Sakaguchi Y Haneda T Mizutani H Yoshimoto T. Skin-specific expression of IL-33 activates group 2 innate lymphoid cells and elicits atopic dermatitis-like inflammation in mice. Proc Natl Acad Sci USA. (2013) 110:13921–6. 10.1073/pnas.1307321110 23918359 PMC3752227

[78] Oldhoff JM Darsow U Werfel T Katzer K Wulf A Laifaoui J. Anti-IL-5 recombinant humanized monoclonal antibody (Mepolizumab) for the treatment of atopic dermatitis. Allergy. (2005) 60:693–6. 10.1111/j.1398-9995.2005.00791.x 15813818

[79] Asherson GL Barnes RMR. Contact sensitivity in the mouse. Immunology. (1973) 24:885–94. 4715261 PMC1422815

[80] Fyhrquist N Wolff H Lauerma A Alenius H. CD8^+^ T cell migration to the skin requires CD4^+^ help in a murine model of contact hypersensitivity. PLoS ONE. (2012) 7:e41038. 10.1371/journal.pone.0041038 22916101 PMC3423415

[81] Martin SF. Allergic contact dermatitis: xenoinflammation of the skin. Curr Opin Immunol. (2012) 24:720–9. 10.1016/j.coi.2012.08.003 22980498

[82] Dudeck A Dudeck J Scholten J Petzold A Surianarayanan S Köhler A. Mast cells are key promoters of contact allergy that mediate the adjuvant effects of haptens. Immunity. (2011) 34:973–84. 10.1016/j.immuni.2011.03.028 21703544

[83] Esser PR Wölfle U Dürr C von Loewenich FD Schempp CM Freudenberg MA. Contact sensitizers induce skin inflammation via ROS production and hyaluronic acid degradation. PLoS ONE. (2012) 7:e41340. 10.1371/journal.pone.0041340 22848468 PMC3405137

[84] Martin SF Dudda JC Bachtanian E Lembo A Liller S Durr C. Toll-like receptor and IL-12 signaling control susceptibility to contact hypersensitivity. J Exp Med. (2008) 205:2151–62. 10.1084/jem.20070509 18725520 PMC2526208

[85] Weber FC Németh T Csepregi JZ Dudeck A Roers A Ozsvári B. Neutrophils are required for both the sensitization and elicitation phase of contact hypersensitivity. J Exp Med. (2015) 212:15–22. 10.1084/jem.20130062 25512469 PMC4291534

[86] Weber FC Esser PR Müller T Ganesan J Pellegatti P Simon MM Lack of the purinergic receptor P2X7 results in resistance to contact hypersensitivity. J Exp Med. (2010) 207:2609–19. 10.1084/jem.20092489 21059855 PMC2989767

[87] Tang L Peng H Zhou J Chen Y Wei H Sun R. Differential phenotypic and functional properties of liver-resident NK cells and mucosal ILC1s. J Autoimmun. (2016) 67:29–35. 10.1016/j.jaut.2015.09.004 26422992

[88] Paust S Gill HS Wang B-Z Flynn MP Moseman EA Senman B. Critical role for the chemokine receptor CXCR6 in NK cell–mediated antigen-specific memory of haptens and viruses. Nat Immunol. (2010) 11:1127–35. 10.1038/ni.1953 20972432 PMC2982944

[89] Rafei-Shamsabadi DA van de Poel S Dorn B Kunz S Martin SF Klose CSN Lack of type 2 innate lymphoid cells promote a type I driven enhanced immune response in contact hypersensitivity. J Invest Dermatol. (2018) 138:1962–72. 10.1016/j.jid.2018.03.001 29526762 PMC6117454

[90] Halim TYF Steer CA Mathä L Gold MJ Martinez-Gonzalez I McNagny KM. Group 2 innate lymphoid cells are critical for the initiation of adaptive T Helper 2 cell-mediated allergic lung inflammation. Immunity. (2014) 40:425–35. 10.1016/j.immuni.2014.01.011 24613091 PMC4210641

[91] Moro K Yamada T Tanabe M Takeuchi T Ikawa T Kawamoto H. Innate production of T(H)2 cytokines by adipose tissue-associated c-Kit(+)Sca-1(+) lymphoid cells. Nature. (2010) 463:540–4. 10.1038/nature08636 20023630

[92] Herrick CA Xu L McKenzie ANJ Tigelaar RE Bottomly K IL-13 is necessary, not simply sufficient, for epicutaneously induced Th2 responses to soluble protein antigen. J Immunol. (2003) 170:2488–95. 10.4049/jimmunol.170.5.2488 12594274

[93] Martin SF Jakob T. From innate to adaptive immune responses in contact hypersensitivity. Curr Opin Allergy Clin Immunol. (2008) 8:289–93. 10.1097/ACI.0b013e3283088cf9 18596583

[94] Lass C Merfort I Martin SF. *In vitro* and *in vivo* analysis of pro- and anti-inflammatory effects of weak and strong contact allergens. Exp Dermatol. (2010) 19:1007–13. 10.1111/j.1600-0625.2010.01136.x 20701630

[95] Dearman RJ Kimber I. Role of CD4^+^ T helper 2-type cells in cutaneous inflammatory responses induced by fluorescein isothiocyanate. Immunology. (2000) 101:442–51. 10.1046/j.1365-2567.2000.01126.x 11122447 PMC2327104

[96] Ogawa A Yoshizaki A Yanaba K Ogawa F Hara T Muroi E. The differential role of L-selectin and ICAM-1 in Th1-type and Th2-type contact hypersensitivity. J Invest Dermatol. (2010) 130:1558–70. 10.1038/jid.2010.25 20182448 PMC3733127

[97] Bao K Yu X Wei X Gui L Liu H Wang X. Astragaloside IV ameliorates allergic inflammation by inhibiting key initiating factors in the initial stage of sensitization. Sci Rep. (2016) 6:38241. 10.1038/srep38241 27917896 PMC5137013

[98] Porcelli S Brenner MB Greenstein JL Terhorst C Balk SP Bleicher PA Recognition of cluster of differentiation 1 antigens by human CD4^−^CD8^−^ cytolytic T lymphocyte. Nature. (1989) 341:447–50. 10.1038/341447a0 2477705

[99] Kim JH Hu Y Yongqing T Kim J Hughes VA Nours JL CD1a on Langerhans cells controls inflammatory skin diseases. Nat Immunol. (2016) 17:1159–66. 10.1038/ni.3523 27548435 PMC5791155

[100] Hardman CS Chen Y-L Salimi M Jarrett R Johnson D Järvinen VJ. CD1a presentation of endogenous antigens by group 2 innate lymphoid cells. Sci Immunol. (2017) 2:eaan5918. 10.1126/sciimmunol.aan5918 29273672 PMC5826589

[101] Mashiko S Mehta H Bissonnette R Sarfati M Increased frequencies of basophils, type 2 innate lymphoid cells and Th2 cells in skin of patients with atopic dermatitis but not psoriasis. J Dermatol Sci. (2017) 88:167–74. 10.1016/j.jdermsci.2017.07.003 28743611

[102] Kim BS Wang K Siracusa MC Saenz SA Brestoff JR Monticelli LA. Basophils promote innate lymphoid cell responses in inflamed skin. J Immunol. (2014) 193:3717–25. 10.4049/jimmunol.1401307 25156365 PMC4170007

[103] Egawa M Mukai K Yoshikawa S Iki M Mukaida N Kawano Y. Inflammatory monocytes recruited to allergic skin acquire an anti-inflammatory M2 phenotype via basophil-derived interleukin-4. Immunity. (2013) 38:570–80. 10.1016/j.immuni.2012.11.014 23434060

[104] Mukai K Matsuoka K Taya C Suzuki H Yokozeki H Nishioka K. Basophils play a critical role in the development of IgE-mediated chronic allergic inflammation independently of T cells and mast cells. Immunity. (2005) 23:191–202. 10.1016/j.immuni.2005.06.011 16111637

[105] Cao Q Wang Y Niu Z Wang C Wang R Zhang Z. Potentiating tissue-resident type 2 innate lymphoid cells by IL-33 to prevent renal ischemia-reperfusion injury. J Am Soc Nephrol. (2018) 29:961–76. 10.1681/ASN.2017070774 29295873 PMC5827602

[106] Besnard A-G Guabiraba R Niedbala W Palomo J Reverchon F Shaw TN. IL-33-mediated protection against experimental cerebral malaria is linked to induction of type 2 innate lymphoid cells, M2 macrophages and regulatory T cells. PLoS Pathog. (2015) 11:e1004607. 10.1371/journal.ppat.1004607 25659095 PMC4450060

[107] Oldenhove G Boucquey E Taquin A Acolty V Bonetti L Ryffel B. PD-1 is involved in the dysregulation of type 2 innate lymphoid cells in a murine model of obesity. Cell Rep. (2018) 25:2053–60.e4. 10.1016/j.celrep.2018.10.091 30463004

[108] Molofsky AB Nussbaum JC Liang H-E Van Dyken SJ Cheng LE Mohapatra A. Innate lymphoid type 2 cells sustain visceral adipose tissue eosinophils and alternatively activated macrophages. J Exp Med. (2013) 210:535–49. 10.1084/jem.20121964 23420878 PMC3600903

[109] Omata Y Frech M Primbs T Lucas S Andreev D Scholtysek C. Group 2 innate lymphoid cells attenuate inflammatory arthritis and protect from bone destruction in mice. Cell Rep. (2018) 24:169–80. 10.1016/j.celrep.2018.06.005 29972778

[110] Obata-Ninomiya K Ishiwata K Tsutsui H Nei Y Yoshikawa S Kawano Y. The skin is an important bulwark of acquired immunity against intestinal helminths. J Exp Med. (2013) 210:2583–95. 10.1084/jem.20130761 24166714 PMC3832932

[111] Naik S Bouladoux N Linehan JL Han S-J Harrison OJ Wilhelm C. Commensal–dendritic-cell interaction specifies a unique protective skin immune signature. Nature. (2015) 520:104–8. 10.1038/nature14052 25539086 PMC4667810

[112] Linehan JL Harrison OJ Han S-J Byrd AL Vujkovic-Cvijin I Villarino AV. Non-classical immunity controls microbiota impact on skin immunity and tissue repair. Cell. (2018) 172:784–96.e18. 10.1016/j.cell.2017.12.033 29358051 PMC6034182

[113] Byrd AL Deming C Cassidy SKB Harrison OJ Ng W-I Conlan S. *Staphylococcus aureus* and *Staphylococcus epidermidis* strain diversity underlying pediatric atopic dermatitis. Sci Transl Med. (2017) 9:eaal4651. 10.1126/scitranslmed.aal4651 28679656 PMC5706545

[114] Ricardo-Gonzalez RR Van Dyken SJ Schneider C Lee J Nussbaum JC Liang H-E. Tissue signals imprint ILC2 identity with anticipatory function. Nat Immunol. (2018) 19:1093–9. 10.1038/s41590-018-0201-4 30201992 PMC6202223

[115] Silver JS Kearley J Copenhaver AM Sanden C Mori M Yu L Inflammatory triggers associated with exacerbations of COPD orchestrate plasticity of group 2 innate lymphoid cells in the lungs. Nat Immunol. (2016) 17:626–35. 10.1038/ni.3443 27111143 PMC5345745

[116] Stehle C Saikali P Romagnani C. Putting the brakes on ILC2 cells. Nat Immunol. (2016) 17:43–4. 10.1038/ni.3353 26681462

[117] Duerr CU McCarthy CDA Mindt BC Rubio M Meli AP Pothlichet J. Type I interferon restricts type 2 immunopathology through the regulation of group 2 innate lymphoid cells. Nat Immunol. (2015) 17:65–75. 10.1038/ni.3308 26595887 PMC9135352

[118] Bi J Cui L Yu G Yang X Chen Y Wan X. NK cells alleviate lung inflammation by negatively regulating group 2 innate lymphoid cells. J Immunol. (2017) 198:3336–44. 10.4049/jimmunol.1601830 28275135

[119] Kim HS Jang J-H Lee MB Jung ID Kim YM Park Y-M. A novel IL-10-producing innate lymphoid cells (ILC10) in a Contact Hypersensitivity Mouse Model. BMB Rep. (2016) 49:293–6. 10.5483/BMBRep.2016.49.5.023 26949018 PMC5070710

[120] Madan R Demircik F Surianarayanan S Allen JL Divanovic S Trompette A Non-redundant roles for B cell-derived IL-10 in immune counter-regulation. J Immunol. (2009) 183:2312–20. 10.4049/jimmunol.0900185 19620304 PMC2772089

[121] Molofsky AB Van Gool F Liang H-E Van Dyken SJ Nussbaum JC Lee J. Interleukin-33 and interferon-γ counter-regulate group 2 innate lymphoid cell activation during immune perturbation. Immunity. (2015) 43:161–74. 10.1016/j.immuni.2015.05.019 26092469 PMC4512852

[122] Rauber S Luber M Weber S Maul L Soare A Wohlfahrt T. Resolution of inflammation by interleukin-9-producing type 2 innate lymphoid cells. Nat Med. (2017) 23:938–44. 10.1038/nm.4373 28714991 PMC5575995

[123] Wohlfert EA Grainger JR Bouladoux N Konkel JE Oldenhove G Ribeiro CH. GATA3 controls Foxp3^+^ regulatory T cell fate during inflammation in mice. J Clin Invest. (2011) 121:4503–15. 10.1172/JCI57456 21965331 PMC3204837

[124] Malhotra N Leyva-Castillo JM Jadhav U Barreiro O Kam C O'Neill NK. RORα-expressing T regulatory cells restrain allergic skin inflammation. Sci Immunol. (2018) 3:eaao6923. 10.1126/sciimmunol.aao6923 29500225 PMC5912895

[125] Cardoso V Chesné J Ribeiro H García-Cassani B Carvalho T Bouchery T. Neuronal regulation of type 2 innate lymphoid cells via neuromedin U. Nature. (2017) 549:277–81. 10.1038/nature23469 28869974 PMC5714273

[126] Galle-Treger L Suzuki Y Patel N Sankaranarayanan I Aron JL Maazi H. Nicotinic acetylcholine receptor agonist attenuates ILC2-dependent airway hyperreactivity. Nat Commun. (2016) 7:13202. 10.1038/ncomms13202 27752043 PMC5071851

[127] Klose CSN Mahlakõiv T Moeller JB Rankin LC Flamar A-L Kabata H. The neuropeptide neuromedin U stimulates innate lymphoid cells and type 2 inflammation. Nature. (2017) 549:282–6. 10.1038/nature23676 28869965 PMC6066372

[128] Moriyama S Brestoff JR Flamar A-L Moeller JB Klose CSN Rankin LC. β2-adrenergic receptor–mediated negative regulation of group 2 innate lymphoid cell responses. Science. (2018) 359:1056–61. 10.1126/science.aan4829 29496881

[129] Sui P Wiesner DL Xu J Zhang Y Lee J Dyken SV. Pulmonary neuroendocrine cells amplify allergic asthma responses. Science. (2018) 360:eaan8546. 10.1126/science.aan8546 29599193 PMC6387886

[130] Talbot S Abdulnour R-EE Burkett PR Lee S Cronin SJF Pascal MA. Silencing nociceptor neurons reduces allergic airway inflammation. Neuron. (2015) 87:341–54. 10.1016/j.neuron.2015.06.007 26119026 PMC4506220

[131] Wallrapp A Riesenfeld SJ Burkett PR Abdulnour R-EE Nyman J Dionne D. The neuropeptide NMU amplifies ILC2-driven allergic lung inflammation. Nature. (2017) 549:351–6. 10.1038/nature24029 28902842 PMC5746044

[132] Liu B Tai Y Achanta S Kaelberer MM Caceres AI Shao X. IL-33/ST2 signaling excites sensory neurons and mediates itch response in a mouse model of poison ivy contact allergy. Proc Natl Acad Sci USA. (2016) 113:E7572–9. 10.1073/pnas.1606608113 27821781 PMC5127381

[133] Zhao Z-Q Huo F-Q Jeffry J Hampton L Demehri S Kim S. Chronic itch development in sensory neurons requires BRAF signaling pathways. J Clin Invest. (2013) 123:4769–80. 10.1172/JCI70528 24216512 PMC3809799

[134] Oetjen LK Mack MR Feng J Whelan TM Niu H Guo CJ. Sensory neurons co-opt classical immune signaling pathways to mediate chronic itch. Cell. (2017) 171:217–28.e13. 10.1016/j.cell.2017.08.006 28890086 PMC5658016

[135] Wilson SR Thé L Batia LM Beattie K Katibah GE McClain SP. The epithelial cell-derived atopic dermatitis cytokine TSLP activates neurons to induce itch. Cell. (2013) 155:285–95. 10.1016/j.cell.2013.08.057 24094650 PMC4041105

